# Effects of Game Situation-Dependent Emotions on Sport Spectators’ Food Craving

**DOI:** 10.3389/fpsyg.2021.724220

**Published:** 2021-11-18

**Authors:** Yonghwan Chang

**Affiliations:** Department of Sport Management, University of Florida, Gainesville, FL, United States

**Keywords:** obesity, uncontrolled eating, affect, emotional coping, spectator sports

## Abstract

This study sought to challenge prevalent accounts of emotional eating by exploring the effects of situation-dependent emotions on consumers’ food craving. Four specific game situations in the context of spectator sports, each corresponding to the four types of emotional coping (outcome-desire conflict, avoidance, fulfillment, and pursuit), were identified as follows: decisive victories, decisive losses, close victories, and close losses. By employing laboratory-based virtual reality spectatorship, Study 1 tested the causal effects of happiness (fulfillment), anger (conflict), sadness (conflict), fear (avoidance), and hope (pursuit) on food craving. Study 2 further designed fans’ previous association between emotions and eating as a moderating mechanism in the context of online sport viewership. The results of the two experiments supported the three theoretical principles of eating behavior, including the “food as fuel” principle of anger, the hedonic eating principle of happiness, and the self-regulation principle of hope. However, the results rejected the escape awareness principle of sadness and fear. The study concludes with a discussion of context-dependent emotional positioning and intervention strategies for marketers and policy makers.

## Introduction

Obesity is becoming more and more prevalent in the United States (van Strien, 2018). Legislators and health professionals have repeatedly looked for possible means to combat obesity given the serious impact that obesity has on individuals’ health (Vainik et al., 2019). In recent years, scholars have made significance advances in their attempt to better understand underlying elements that must be tackled to reduce the harmful consequences associated with obesity (Sharpe et al., 2008). Notably for the scope of this study, research in the field of consumer behavior and food studies has suggested that individuals’ eating behavior is largely affected by their emotions (van Strien, 2018; Wiedemann et al., 2018; Low et al., 2021). That is, it has been increasingly suggested that emotional eating (caused in response to affective cues) contributes to uncontrolled eating, overeating, and obesity (Nicholls et al., 2015; van Strien, 2018; Vainik et al., 2019).

Emotional eating generally refers to the tendency to eat in response to affective states (Arnow et al., 1995; Ferrell et al., 2020; Barnhart et al., 2021). The evidence of affective states as an antecedent to emotional eating has been firmly established (Barnhart et al., 2020). Nonetheless, recent research (e.g., Altheimer and Urry, 2019; Barnhart et al., 2021) has called attention to the suggestion that existing studies on emotional eating have excessively relied on the assumption that negative emotions (rather than positive ones) inherently induce food intake. This conventional belief is rooted in the escape awareness principle of eating.^1^ As such, the general assumption is that emotional consumers are often involved in this habitual and compulsive consumption to deal with unfavorable emotions (Macht, 1999). This presumption, however, has been counter-argued by the notion that positive emotions have also the potential to trigger emotional eating (e.g., Bongers et al., 2013; van Strien, 2018; Altheimer and Urry, 2019).

For example, recent evidence indicates that food craving^2^ can be understood as a learned behavior shaped through cumulative emotional coping experiences (Altheimer and Urry, 2019). That is, individuals often crave food intake under situations that evoke particular emotions because they have associated emotions with eating in the past as a means to address situation-dependent emotional responses (Nath et al., 2020; Low et al., 2021). To illustrate, sport fans often react emotionally to the performance of athletes/teams they support while also frequently conducting an emotional assessment of events as they watch a game (Cornil and Chandon, 2013). Specific game situations may then activate spectators’ intensive emotional experiences, potentially contributing to and shaping their eating tendencies. For example, the game situation of close losses may cause fans to experience feelings of sadness or anger and to undoubtedly activate the coping process of outcome-desire conflict to deal with the loss of their favored team (Geniole et al., 2017). On the other hand, the game situation of decisive victories is likely to contribute to fans’ feelings of happiness as well as activate their coping experiences of outcome-desire fulfillment given that, in this scenario, fans’ goals are achieved, and the outcomes are pleasurable (Mehta et al., 2015).

Fans may then crave food intake to alleviate their negative feelings and shift attention away from disappointing situations (i.e., the escape awareness principle of sadness; Macht, 1999), to fuel their bodily and psychic energy (i.e., the “food as fuel” principle of anger; Cornil et al., 2020), or to augment their pleasurable feelings (i.e., the hedonic eating principle of happiness; Salerno et al., 2014). In the meantime, the nuanced game situation of close losses is likely to also concurrently evoke feelings of hope (in addition to negative feelings such as anger); that is, the fans of that team may remain hopeful and pursue positive outcomes in the future, presumably resulting in aversive effects on their eating habits (i.e., the self-regulation principle of hope; Reynolds and McCrea, 2017; Stautz et al., 2018).

As such, game situations-specific emotions are likely to interact with the way spectators have learned to associate particular emotions with eating in the past, affecting the dynamics that determine their food consumption. Based on this understanding, the current study aims to examine the effects of situation-dependent spectator emotions on food craving in conjunction with previously established associations between emotions and eating.

## Theoretical Background and Hypotheses Development

### Emotional Coping and Food Craving

Emotion theorists have suggested that emotions are discrete, with each type of discrete emotions involving subjective experiences of psychophysiological changes that result in unique behavioral reaction patterns (Macht, 1999). For example, sadness is a type of negative affect that largely induces a sense of loss, helplessness, and disappointment (Salerno et al., 2014); therefore, an overreaching adaptive response of sadness is self-verification (such as seeking out social acceptance and self-promotion; Macht, 1999). On the other hand, happiness signals success and achievement (Bongers et al., 2013), which is why happy people tend to be perceived as being more socially attractive (Tanzer and Weyandt, 2019). As a result, happy people often seek to continue feeling the emotion of happiness and even desire to augment the pleasurable feelings they are currently experiencing in their happy state (Bongers et al., 2013).

The emotional coping account of eating (e.g., Okumus and Ozturk, 2021) asserts that emotions play a central role in regulating food intake because eating is a product of a series of coping processes that allow humans to control fluctuating mood states. According to the sequential processes of emotional coping (Cornil et al., 2020), individuals often appraise the outcomes of an event by comparing them to a desired state of emotional experiences. As a result of the outcome-desire appraisal process, these individuals experience either positively or negatively valenced affective states (Bagozzi, 1992). Certain behavioral actions, such as eating, are then adapted and implemented as coping responses corresponding to the aroused affective states (Salerno et al., 2014). For example, in response to outcome-desire conflicts/disparities or goal failures (i.e., situations that evoke negative emotions such as sadness and anger), individuals often crave sweet and savory foods to direct their temporal affective states toward a desired outcome. Moreover, research suggests that food intake is generally an accessible option to shift one’s attention away from negative emotions-evoking events (Reynolds and McCrea, 2017; Barnhart et al., 2020).

Based on this background knowledge, emotion theorists (e.g., Bagozzi, 1992; Winterich and Haws, 2011) have further illustrated these sequential processes by identifying the four types of emotional coping: (1) outcome-desire conflict, (2) outcome-desire avoidance, (3) outcome-desire fulfillment, and (4) outcome-desire pursuit. In the context of spectator sports, the unscripted performance and outcomes of a sport event lend themselves to an evolving emotional experience (Campo et al., 2019). Fans’ emotions are then arguably largely influenced by the final outcome of a game as well as the dynamics of a games’ processes and various events that transpire throughout a game (Mehta et al., 2015). As such, fans’ emotional coping responses correspond to the specific game situations of the team/athlete fans support when they are in the role of spectators. In addition, appraisal theories in psychology suggest that emotions and emotional coping responses arise following individuals’ evaluation and interpretation of certain events (Salerno et al., 2014; Cornil et al., 2020). The following sections delineate how the four types of emotional coping correspond to particular game situations in the context of spectator sports.

#### Outcome-Desire Conflict

Outcome-desire conflicts happen when people fail to accomplish objectives or experience unfavorable situations (Bagozzi, 1992); therefore, this type of appraisal is likely to induce negatively valenced emotions such as anger and sadness (Tiedens, 2001). The primary coping response to outcome-desire conflicts is seeking to reduce harm (Bagozzi, 1992). Studies on emotional coping (e.g., Mikulincer and Shaver, 2019) suggest that an increase in anger and sadness triggers emotional eating in an attempt to reduce negative emotions by providing a temporary distraction or sense of comfort (Overall et al., 2015).

In the context of spectator sports, the game situations of close and decisive losses are likely to induce spectators’ appraisals of outcome-desire conflict. Research shows that, generally, both types of discrete emotions – anger and sadness – are similarly triggered by experiencing loss, failure, and defeat (Barnhart et al., 2020; Chang and Inoue, 2021); consequently, feelings of anger and sadness concurrently emerge in both game situations involving the team’s loss simply because fans’ favored team was defeated and unsuccessful. However, anger often arises when goals are thwarted but remain potentially attainable (e.g., a close loss), while sadness is likely to elicit a sense of loss when goals appear beyond reach (e.g., decisive loss; Weisberg et al., 2016). Regardless of the specific types of negative emotions, as the emotional coping account of eating (e.g., Salerno et al., 2014; Cornil et al., 2020) suggests, both anger and sadness are likely to induce spectators’ food craving in an attempt to alleviate the negative feelings experienced by spectators.

*Hypothesis 1A*: Sport spectators’ outcome-desire conflict-induced anger positively influences their food craving.

*Hypothesis 1B*: Sport spectators’ outcome-desire conflict-induced *sadness positively* influences their food craving.

#### Outcome-Desire Avoidance

Both outcome-desire conflict and avoidance induce negatively valenced emotions on their own. Outcome-desire avoidances, however, differ from outcome-desire conflicts in regard to temporal focus (i.e., future- vs. past- or present-focused; Winterich and Haws, 2011). With respect to outcome-desire avoidances, the appraisal is largely based on the anticipation of an unpleasant future event (rather than after the event already took place as is the case for outcome-desire conflicts; Bagozzi, 1992). Fear, worry, and anxiety are emotions that primarily arise from outcome-desire avoidances, with the coping response aiming to avoid undesirable future outcomes (Bagozzi et al., 1999). Individuals experience fear and anxiety when their surrounding situations are uncertain, cautious, and seemingly threatening (Dunn and Hoegg, 2014). Fear promotes avoidant behaviors (Madrigal and Bee, 2005); thus, individuals tend to cope with the intent to avoid the unfavorable consequence or reinterpret the potential danger or risk with outcome-desire avoidance appraisals (Mikulincer and Shaver, 2019).

Individuals often increase food consumption in their attempt to regulate negative emotions that have an avoidance motivational component (Macht, 1999). The escape awareness principle included in the emotional coping account (Reynolds and McCrea, 2017; Barnhart et al., 2020) suggests that people overeat in response to negative emotions resulting from a desire to escape or stay away from an unpleasant stimulus. Thus, eating becomes a tool to avoid negative thoughts and emotions. In the context of spectator sports, fans may perceive the close victories of a favored team as a situation where the winning position is insecure, susceptible, and illegitimate because said victories were hardly attained (Mehta et al., 2015). Consequently, fear arises due to fans’ concern over the close possibility of losing; thus, in order to obtain relief from this fear, a sport spectator witnessing a close victory outcome is likely to show an increase in food intake.

*Hypothesis 2*: Sport spectators’ outcome-desire avoidance-induced *fear positively* influences their food craving.

#### Outcome-Desire Fulfillment

In contrast to outcome-desire conflicts and avoidances, outcome-desire fulfillment and outcome-desire pursuits both induce positively valenced emotions (Bagozzi, 1992). Outcome-desire fulfillment occurs when an individual accomplishes objectives and experiences favorable situations (Bagozzi et al., 1999). The main emotion that arises with this appraisal type is happiness (Tanzer and Weyandt, 2019). Outcome-desire fulfillment transpires when an individual accomplishes objectives, experiences favorable situations, or escapes from unfavorable situations, thus invoking happiness (Bongers et al., 2013). When experiencing happiness, individuals cope with the intent to maintain, increase, or share the outcome (Bagozzi et al., 1999).

With close and decisive victory situations for a favored team in the context of spectator sports, fans are likely to experience happiness simply because their favored team won (Chang and Inoue, 2021). While the majority of research developed based on the emotional coping account of eating has highlighted food intake as a mechanism to relieve unfavorable emotions, research has shown that favorable affect can also result in increased emotional eating (Cardi et al., 2015). For example, studies on hedonic eating (e.g., Bongers et al., 2013; Salerno et al., 2014) suggest that happy consumers are likely to show a strong desire for food intake in an attempt to sustain and augment their positive feelings. Accordingly, it is expected that sport spectators experiencing outcome-desire fulfillments due to their favored team’s victory will turn to eating as a means to celebrate their victory as well as further strengthen their current happiness.

*Hypothesis 3*: Sport spectators’ outcome-desire fulfillment-induced happiness positively influences their food craving.

#### Outcome-Desire Pursuit

Outcome-desire pursuits arise in expectancy of a favorable goal or outcome, which then invokes hope or hopefulness (Bagozzi, 1992). Hope is a positive emotion that arises from thinking about a future desired outcome (Tong and Jia, 2017). Though outcome-desire pursuits are similar to outcome-desire fulfillment in that both types of pursuits induce a positively valenced emotion (i.e., happiness as hypothesized in *H3*), outcome-desire pursuits differ slightly from outcome-desire fulfillment in that the former has a future temporal focus (whereas the latter focuses on the past/present; Winterich and Haws, 2011). That is, outcome-desire pursuits center future positive emotions elicited in response to an anticipated favorable event (Bagozzi et al., 1999). Hopeful individuals often remain assured that their goals can be realized (Tong and Jia, 2017), thus allowing them to cope with the intent to facilitate outcome attainment and sustain commitment toward desired future states (Gao et al., 2016). Hope has also been found to be positively associated with self-control (Tong and Jia, 2017). Individuals with future-focused hopefulness have been found to have greater self-control than individuals who rely on past-focused positive emotions (e.g., happiness; Winterich and Haws, 2011). Taken together, research shows that individuals who feel hopeful (positive emotion accompanied by an emphasis on the future) display lower levels of food consumption than when experiencing happiness or enjoyment (positive emotions accompanied by an emphasis on the past/present; Godfrey et al., 2015; Stautz et al., 2018).

In the context of spectator sports, the game situation of close losses may also induce hope (in addition to anger and sadness experienced through the outcome-desire conflicts appraisal; *H1*). Fans’ perception of close losses may lead them to feel hopeful for a future positive outcome given that, in fans’ minds, such disappointing outcomes were nearly prevented, and the favored team’s relatively lower position becomes perceived as tentative and illegitimate (Mehta et al., 2015). Furthermore, sport spectators’ future-focused feelings of hopefulness are likely to help enhance positivity attributed to (i) past memories of victories and successes, (ii) finding more important meanings (e.g., team tradition) in unfavorable circumstances, and (iii) guarding against the arousal of negative emotions in the future. As such, it is predicted that hopeful sport spectators coping with the game outcomes of close losses may not necessarily need to cope through emotional eating mechanisms; rather, feelings of hope may optimistically affect fans’ self-regulation capability by associating their current consumption choices with future goal attainment, resulting in aversive effects on food craving.

*Hypothesis 4*: Sport spectators’ outcome-desire pursuit-induced hope negatively influences their food craving.

## Experiment 1

### Design and Procedure

Seventy undergraduate students were recruited at a large midwestern university; a majority of the participants was Caucasian (*n*=61, 88%) and male (*n*=50, 72%), with an average age of 21.97years (*SD*=1.95). Randomly intercepted samples at university buildings who agreed to participate in this study were led to the experimental laboratory. They were randomly assigned to a basketball game played in the 2019–2020 National Basketball Association (NBA) season and then were prompted to watch the game for approximately 15min by using the Oculus Go virtual reality device^3^ provided to them on site. Immediately following the viewing task, participants were asked to respond to a survey where they rated the extent to which they experienced the emotional states of hope (“*hopeful*,” “*optimistic*,” and “*positive*”), anger (“*angry*,” “*annoyed*,” and “*irritated*”), fear (“*fearful*,” “*scared*,” and “*anxious*”), sadness (“*sad*,” “*gloomy*,” and “*blue*”), and happiness (e.g., “*happy*,” “*pleased*,” and “*delighted*”) while watching the game, ranging from 1=not at all to 7=extremely (Coleman and Williams, 2013; Chang et al., 2018).

They were also asked to respond to the measure of food craving [“Indicate the extent to which you feel an urge to eat (one or more specific foods)”: 1=I have no desire to eat, 7=I have an overwhelming urge to eat; “I am craving (one or more specific foods)”: 1=strongly disagree, 7=strongly agree; “If I had (one or more specific foods), I could not stop eating it”: 1=strongly disagree, 7=strongly agree; Arnow et al., 1995]. The covariate measures included NBA fanship (“I am a fan of NBA games”: 1=strongly disagree, 7=strongly agree) and Team identification (“I identify myself as part of the team”: 1=strongly disagree, 7=strongly agree). The last item in the survey asked participants to respond to the measure of perceived decisiveness/closeness of a game (“Overall, the game was decisive/close”: 1=decisive game, 7=close game). In addition, as part of the survey, participants were asked to provide a brief written report about the game they viewed, including the team they supported and the final game result; in this report, they were also asked to complete a categorization task about the game they viewed (“My team had a”: 1=decisive victory, 2=decisive loss, 3=close victory, 4=close loss). Upon successful completion of the experiment, they were thanked and compensated with a $10 prepaid debit card.

### Results

The participants’ game situations categorization task was reviewed through their open-ended responses to check for manipulations of the game situations. Specifically, the section of the open-ended responses was inspected where participants identified the name of the team each of them supported, and the final score of the game the participant at hand was tasked with viewing; this information was then matched with each participant’s response to the situation categorization task. The results showed no violated matches.

As a supplement, after controlling for the two covariates, the ANCOVA results revealed significant differences between the two conditions: close vs. decisive [*F*(1, 66)=6.83, *p*=0.01]; specifically, the two close games were perceived to be more competitive (i.e., closer) than the decisive ones (*M*_close games_=4.56 vs. *M*_decisive games_=3.81). The multiple items assessing hope (*α*=0.92), anger (*α*=0.91), fear (*α*=0.82), sadness (*α*=0.92), happiness (*α*=0.95), and food craving (*α*=0.87) were averaged to form a composite variable for each construct, respectively.

The ANCOVA results revealed a series of dynamics that have significant main effects on the five different types of discrete emotions. Specifically, an inspection of the least-squares adjusted means (LSM) for each discrete emotion indicated the four different game situations had significant main effects on happiness [*F*(3, 64)=3.93, *p*=0.01], anger [*F*(3, 64)=5.55, *p*=0.002], and hope [*F*(3, 64)=3.99, *p*=0.01]; on the other hand, sadness and fear were not significantly different across the four different game situations. In terms of the outcome-desire conflicts assumption, anger was the most blatant emotion category in the close loss condition. Furthermore, a regression analysis revealed that feelings of anger evoked in both game conditions, close loss (*β*=0.62, *SE*=0.27, *p*=0.002) and decisive loss (*β*=0.47, *SE*=0.18, *p*=0.02), significantly and positively influenced food craving. The results supported *H1A*.

**Figure 1:**
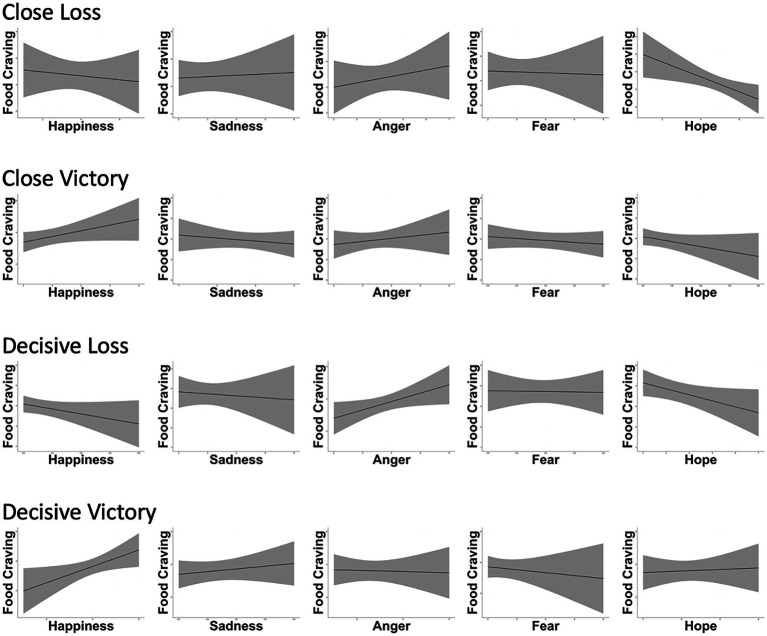
A summary of the Experiment 1 results: casual influences that game situation-dependent discrete emotions exert on sport spectators’ food craving.

In addition to anger, close losses condition led to the highest scores in levels of hope; feelings of hope in this condition significantly and negatively affected food craving (*β*=−0.39, *SE*=0.29, *p*=0.04). Accordingly, the results support the assumption that sport spectators’ outcome-desire pursuit-induced hope negatively influences their food craving (*H4*). With respect to outcome-desire fulfillments, decisive victories resulted in the happiest state among spectators. Both decisive victories (*β*=0.84, *SE*=0.28, *p*=0.01) and close victories (*β*=0.29, SE=0.20, *p*=0.04) exerted significantly positive influences of happiness on food craving, thus supporting *H3*. Both feelings of sadness and fear revealed non-significant effects on food craving in all of the four game situations, rejecting *H1B* and *H2* (Figure 1 and Table 1).

**Table 1:** Correlation coefficients, F-statistics (Sig.), and raw means (least square adjusted means; LSMs).

	1	2	3	4	5	6	7	8
1. Happiness	1.00							
2. Sadness	0.08	1.00						
3. Anger	0.32^**^	0.19	1.00					
4. Fear	0.12	0.30^**^	0.34^**^	1.00				
5. Hope	0.33^**^	−0.05	−0.08	0.03	1.00			
6. Food craving	0.39^***^	0.15	0.46^***^	0.17	−0.26^*^	1.00		
7. Team ID	0.19	−0.04	0.32^**^	0.13	0.28^*^	0.19	1.00	
8. NBA Fanship	0.15	−0.01	0.36^**^	−0.02	−0.04	0.11	0.24^*^	1.00
	Happiness	Sadness	Anger	Fear	Hope	Food Craving		
Game situations	3.93^*^ (0.01)	0.59 (0.63)	5.55^**^ (0.002)	1.27 (0.29)	3.99^*^ (0.01)	0.92 (0.44)		
NBA Fanship	0.91 (0.34)	0.04 (0.84)	4.66^*^ (0.03)	1.44 (0.23)	1.48 (0.23)	1.09 (0.29)		
Team ID	1.03 (0.32)	0.05 (0.83)	8.58^**^ (0.005)	0.72 (0.40)	4.32^*^ (0.04)	2.39 (0.13)		
Decisive Victory	3.94 (4.11)^a^	2.67 (2.64)	2.67 (3.04)	2.56 (2.47)	4.17 (4.11)	4.11 (4.31)		
Decisive Loss	2.94 (2.99)	3.06 (3.05)	3.78 (3.91)	2.94 (2.97)	3.44 (3.53)	3.56 (3.63)		
Close Victory	4.29 (4.09)	2.71 (2.74)	3.41 (2.99)	2.94 (2.98)	4.29 (4.26)	4.06 (3.83)		
Close Loss	3.71 (3.66)	2.94 (2.95)	4.12 (4.01)^b^	3.12 (3.14)	4.82 (4.83)^c^	3.71 (3.65)		

* *p*<0.05;

** *p*<0.01;

*** *p*<0.001.

a Significantly greater than Decisive Loss.

b Significantly greater than Close Victory.

c Significantly greater than Decisive Loss.

### Discussion

Experiment 1 tested causal influences that game situation-dependent discrete emotions exert on sport spectators’ food craving by applying the four types of emotional coping. In summary, the results supported the emotional coping processes of outcome-desire conflict (anger), fulfillment (happiness), and pursuit (hope) in the given context; however, the results did not support the emotional coping processes of outcome-desire conflict (sadness) and avoidance (fear). The present results, therefore, suggest that anger has a stronger influence on emotional eating and food craving than other types of negative emotions, such as sadness and fear. One compelling account of the positive association between negative emotions and emotional eating is the escape awareness theory of emotions. Quite simply, the escape awareness theory (e.g., Macht, 1999; Barnhart et al., 2020) asserts that under stressful situations eliciting unfavorable feelings, individuals often desire to eat as a means to shift their attention away from negative emotions-eliciting stimuli (i.e., cognitive narrowing; Barnhart et al., 2020). That is, food intake functions to mask individuals’ immediate and negative feelings of irritation, anxiety, and sadness (Reynolds and McCrea, 2017). However, given the results, the escape awareness is not well supported in the context of spectator sports.

Alternatively, the “food as fuel” mechanism is supported by the results. Scholars in consumer psychology and behavioral science (e.g., Cornil et al., 2020) have recently suggested that consumers tend to perceive food as an energy source and fuel for the body as well as the mind. In spite of not knowing the specific nutritional and caloric information of a food product, food (even unhealthy and high-caloric food) is generally perceived to help boost bodily and psychic energy (Bongers et al., 2013). The “food craving as fuel” phenomenon is, thus, prominent, especially when performance-related goals are activated (Cornil et al., 2020). In this regard, the contextual aspect of spectatorship sports (i.e., athletic performance-related) and the feelings of anger (evoked though experiencing close and suspenseful defeat from others) are likely to largely activate the “food as fuel” mindset as well as emotional eating. In sum, the “food as fuel” mechanism better accounts for sport fans’ emotional eating than the escape awareness account (i.e., shifting their attention away from negative emotions-eliciting stimuli; cognitive narrowing in the situations of sadness and fear).

While the majority of emotional eating research has largely focused on negative emotions, it has been increasingly suggested that positive emotions (such as joy and excitement) have the potential to trigger emotional eating (Barnhart et al., 2020). For example, research suggests that emotional eating triggered by positive emotions happens with the same frequency as emotional eating triggered by negative emotions (Bongers et al., 2013; Vainik et al., 2019). The reasoning behind this theory is that the feeling of heightened pleasantness caused by eating appetizing food is the same regardless of individuals’ prior emotional valence (Salerno et al., 2014; Wiedemann et al., 2018). In brief, these studies all point to increased food consumption as a product of experiencing positive emotions. In line with these recent findings, the results of Experiment 1 revealed that happiness evoked through experiencing decisive victories induced food craving.

The results revealed that the feelings of hope are not associated with emotional eating; the close loss condition even resulted in hope having aversive effects on food craving. Although there is lack of theoretical knowledge behind this relationship, the functionalist perspective of emotions may account for the negative consequences of hope on emotional eating. Existing studies (e.g., Griskevicius and Kenrick, 2013; Luong et al., 2016) suggest that negative emotions (e.g., anger) often facilitate the active use of working memory, resulting in ego depletion and low self-control; on the other hand, certain types of positive emotions (e.g., hope) help preserve individuals’ working memory capacity as well as their cognitive control. That is, working memory is a memory facility containing important information in a short term (Ferrell et al., 2020); when having to face a challenging situation (e.g., close loss in spectatorship sports), individuals with higher working memory capacity have been found to be more likely to effectively manage situations-evoking emotions (i.e., high self-control; Stautz et al., 2018). Furthermore, in their meta-analytic reviews, Moher et al. (2009) found that low self-control often results in increased consumption of alcohol and tobacco as well as uncontrolled eating. Gheller et al. (2019) examined the causal influences of video game playing (VGP) on emotions, appetite, and food intake. They found that negative emotions evoked after VGP positively influenced food intake and subjective appetite; on the other hand, participants who felt positive emotions after VGP desired to eat less, demonstrating high self-control and the ability to resist palatable foods.

Despite such interesting and important implications, several limitations should be addressed. First, individual characteristics might have played a role on the relationships examined. In particular, recent research on food psychology (Altheimer and Urry, 2019) suggests the conjunctional role of previous association between emotion and eating; that is, eating triggered by emotions may require a condition in which individuals have associated emotions with food intake in the past in a given context. For example, in the context of spectator sports, past memories of food consumption while watching a game may largely influence fans’ eating behaviors and food craving. Second, Experiment 1 utilized virtual reality technology as a means to mimic real-life spectating experiences by augmenting fans’ emotional experiences and intensity. Although virtual reality spectatorship is becoming an emerging trend in sport consumption, a criticism of the study could be that the devices associated with VR technology might either have excessively intensified (e.g., sound and visual effects) or suppressed (e.g., dizziness and nausea) spectators’ emotions (Cowan and Ketron, 2019). Experiment 2, then, addresses these potential issues to account for the confounding effects of VR in Experiment 1. Moreover, based on the results of Experiment 1, Experiment 2 was designed to give more attention to the focal discrete emotions of happiness, anger, and hope given the two game situations of decisive victory and close loss.

## Experiment 2

The conditioning framework (Greenwald et al., 2009) accounts for the effects of previous association between emotion and eating. That is, food consumption as an unconditioned stimulus (UCS) is likely to induce an unconditioned response (UCR) such as mood recovery and emotional equilibrium (Hsu and Forestell, 2021); over time, particular discrete emotions (e.g., anger) may have systematically paired with food consumption, engendering emotions to become conditioned stimuli (CS), inducing such conditioned responses as eating (CR; Vainik et al., 2019). According to relevant scholarship in the field, many emotional eaters are unable to distinguish between food craving inflicted by emotions and food craving inflicted by physiological hunger due to their cumulative conditioning experiences (Altheimer and Urry, 2019). Similarly, in the context of spectator sports, past memories of food consumption while watching a game have been found to largely influence fans’ eating behaviors (Cornil and Chandon, 2013). For example, angry fans may attempt to alleviate their feelings of stress with food and beverages while watching suspenseful or unsuccessful games; happy fans, on the other hand, may learn which behaviors induce a positive feeling, such as increased food intake, to celebrate victories or to sustain/augment positive feelings. The learning (or conditioning) processes of mood recovery (or augmentation) through food consumption are likely to have then reinforced fans’ tendencies toward emotional eating. Accordingly, the following hypothesis was formulated.

*Hypothesis 5*: Spectators’ previous association between emotions and eating moderates the relationship between their game situations-evoked emotions and food craving; that is, spectators who have previous associations between emotions and eating are more likely to desire to cope with game situations-evoked emotions through food consumption.

### Design and Procedure

One hundred and five undergraduate and graduate students were recruited from a large US university, 87 of which were male (83%). The average age of the participants was 23.14years (SD=2.45), and a majority of the participants was Caucasian (*n*=65, 62%). The invitation for the online experiment was distributed through instructors at the university, and participants received course credits upon successful completion of the experiment. Pre-screening qualifications included fans’ reported favorability toward the target NBA team to some degree (from mere awareness to fanatical). As proxy measures, this study utilized previous association between emotions and eating (“I often use food to cope with my emotions”: 1=no, 2=yes; “I usually eat more when I’m happy”: 1=strongly disagree, 7=strongly agree; “I tend to eat more when I am upset”: 1=strongly disagree, 7=strongly agree). Then, they were randomly allocated to one of the two game situations, including decisive victory [Timberwolves (winner, 132 pts.) vs. Kings (loser, 105 pts.)] and close loss [Timberwolves (loser, 106 pts.) vs. Nuggets (winner, 107 pts.)] of the team. They were asked to watch a summary of the randomly allocated game extracted from YouTube.com for approximately 15–20min through their own electronic viewing devices (such as laptops or smartphones). The remaining procedures were identical to those in Experiment 1.

### Results

The participants were self-categorized into low vs. high (in relation to their previous association between emotions and eating) based on a binary measure (no vs. yes). The validity of the self-selected categorization was checked by using the two emotional eating scale items (i.e., happy and angry eating). The ANOVA results revealed significant differences between the two conditions; participants placed in the high previous association category showed significantly greater levels of emotional eating [*F*(1, 103)=10.99, *p*=0.001. *M*_low_=3.29, *M*_high_=4.16 for happy eating; *F*(1, 103)=5.39, *p*=0.02. *M*_low_=3.94, *M*_high_=4.46 for angry eating]. Game situations manipulations were checked using the same methodology used in Experiment 1, with the results revealing no violated matches. The ANOVA results further revealed significant differences between the close vs. decisive conditions; the close game was perceived to be more competitive (i.e., closer) compared to the decisive game [*F*(1, 103)=8.29, *p*=0.005; *M*_close game_=4.59 vs. *M*_decisive game_=3.92].

**Figure 2:**
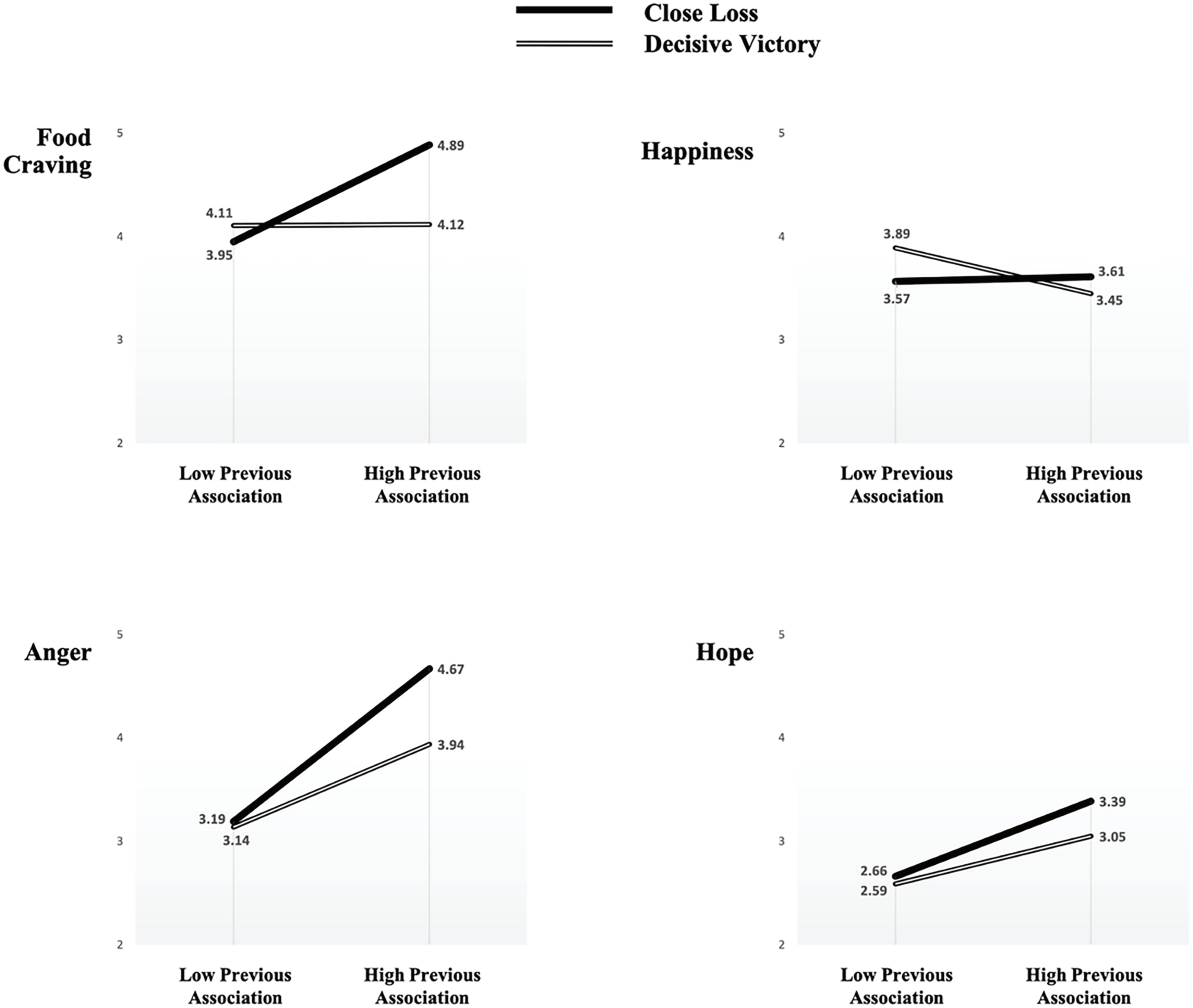
A summary of the 2 (previous association between and eating: low vs. high)×(game situation: close loss vs. decisive victory) results in Experiment 2.

The ANCOVA results revealed significant effects the interactions between game situations and previous association exerted on food craving [*F*(1, 99)=4.32, *p*=0.04]. Meanwhile, game situations had significant main effects on anger [*F*(1, 101)=16.47, *p*<0.001] and food craving [*F*(1, 101)=7.68, *p*=0.007]; specifically, the close loss condition exerted more intensive feelings of anger (*M*_close loss_=4.14 vs. *M*_decisive victory_=3.32, *p*<0.001) as well as greater tendencies toward food craving (*M*_close loss_=4.57 vs. *M*_decisive victory_=3.98, *p*<0.001) compared to the scores in the decisive victories condition. The results also revealed previous association’s significant main effects on anger [*F*(1, 101)=17.73, *p*<0.001], hope (*F*(1, 101)=8.58, *p*=0.004), and food craving [*F*(1, 101)=10.18, *p*=0.002]. That is, the higher levels of sport fans’ previous association, the greater the fans experience feeling anger (*M*_close loss_=4.16 vs. *M*_decisive victory_=3.29, *p*<0.001) and hope (*M*_close loss_=3.18 vs. *M*_decisive victory_=2.67, *p*=0.001) as well as the more they desire to eat (*M*_close loss_=4.02 vs. *M*_decisive victory_=4.50, *p*=0.03). Furthermore, in individuals with high levels of previous associations between emotions and eating, both feelings of happiness (*β*=0.48, *SE*=0.27, *p*=0.03) and anger (*β*=0.31, *SE*=0.11, *p*=0.003) significantly and positively influenced food craving. On the other hand, for individuals with low levels of previous associations between emotions and eating, happiness (*β*=0.29, *SE*=0.21, *p*=0.001) was a sole predictor of food craving. These results support the assumption that spectators who have previous associations are more likely to desire to cope with game situations-evoked emotions through food consumption (Figure 2 and Table 2).

**Table 2:** Correlation coefficients (low/high previous association), F-statistics (Sig.), and LSMs.

	1	2	3	4	5	6
1. Happiness	1.00					
2. Anger	−0.04/0.47^***^	1.00				
3. Hope	−0.01/0.11	0.16/0.34^**^	1.00			
4. Food craving	0.62^***^64^***^	0.01/0.54^***^	−0.07/0.10	1.00		
5. Team ID	0.06/0.35^**^	0.19/0.73^***^	0.18/0.28^*^	0.02/0.57^***^	1.00	
6. NBA Fanship	−0.04/0.12	0.35^**^/0.57^***^	0.16/0.22	0.23^*^/0.29^*^	0.07/0.52^***^	1.00
		Happiness	Anger	Hope	Food Craving	
Game situations		0.003 (0.95)	16.47^***^ (<0.001)	3.41 (0.07)	7.68^**^ (0.007)	
Previous association		1.54 (0.22)	17.73^***^ (<0.001)	8.58^**^ (0.004)	10.18^**^ (0.002)	
Team ID		6.25^*^ (0.01)	39.98^***^ (<0.001)	5.14^*^ (0.03)	12.37^***^ (<0.001)	
NBA Fanship		0.05 (0.82)	11.37^**^ (0.001)	0.71 (0.41)	1.08 (0.31)	
Game Situations×Previous association		0.86 (0.36)	2.42 (0.12)	0.55 (0.46)	4.32^*^ (0.04)	
High previous sssociation	Close loss	3.61	4.67^a^	3.39^b^	4.89^c^	
	Decisive victory	3.45	3.94	3.05	4.12	
Low previous association	Close loss	3.57	3.19	2.66	3.95	
	Decisive victory	3.89	3.14	2.59	4.11	

* *p*<0.05;

** *p*<0.01;

*** *p*<0.001.

a Significantly greater than Close Loss_low Association_ and Decisive Victory_low Association_.

b Significantly greater than Close Loss_low Association_ and Decisive Victory_low Association_.

c Significantly greater than Close Loss_low Association._

## General Discussion

### Theoretical Implications

Past emotion research on eating behavior has revealed considerable inconsistencies across its key findings (Barnhart et al., 2020). As discussed in Introduction, such inconsistencies likely stem from the tendency in most emotional eating studies to predominantly focus on negative emotions, an approach based on the widespread assumption that, given individuals’ inherent desire to avoid harm, negative emotions automatically induce eating (Macht, 1999). Recent studies (e.g., Altheimer and Urry, 2019; Nath et al., 2020), however, have suggested that individuals’ emotional eating might be a behavioral tendency learned from their cumulative socialization process rather than being an inherent characteristic. These studies have further gone on to suggest that some positive emotions could ironically induce food craving and emotional eating behavior (e.g., hedonic and happy eating phenomena; Bongers et al., 2013; Salerno et al., 2014). In line with these keys, more up-to-date findings and in response to the call for more empirical examinations of such recent inquires (Altheimer and Urry, 2019), attempts were made to challenge the prevalent account of emotional eating by exploring the effects of situations-dependent emotions on food craving in the context of spectator sports.

Experiment 2 tested the moderating effects of spectators’ previous association between emotions and eating behavior. The results revealed that spectators who have a previous association are more likely to desire to turn to emotional eating to cope with game situations-evoked feelings. In addition, for spectators who have a previous association, anger was significantly associated with food craving. Research suggests that individuals often desire to eat to help boost bodily and psychic energy as a result of activated performance-related goals (i.e., the “food as fuel” phenomena; Cornil et al., 2020). Research suggests that emotional eating is likely to be more prominent when individuals have associated specific affect with food intake in the past (Wiedemann et al., 2018); in particular, negative emotions, such as anger, are likely to trigger emotional eating as a means to satisfy individuals’ psychological hunger (regardless of their physiological and nutritional emptiness; Vainik et al., 2019). For example, based on a meta-analysis exploring the influence of emotions on food consumption (Godfrey et al., 2015), anger was found to be a prominent antecedent of binge eating and obesity.

It is worth noting that happiness was positively associated with both types of spectators, those who have and those who have not had a relationship between affect and food consumption in the past. The knowledge regarding associations between positive emotions and emotional eating in the existing literature presumably remains limited due to the continued neglect of positive emotions as a risk factor of emotional eating in both therapeutic and real-world settings. For example, Nicholls et al. (2015) suggested that binge eating as a product of positive emotions (e.g., happiness) seldom results in problematic consequences (such as night eating syndrome, severe obesity, and harmed life satisfaction). Barnhart et al. (2021) also argued that humans tend to overeat without emotional triggers because evolution encourages individuals to persist even in times of acute food shortages, leading people to inherently desire to overeat (when food is available) as an adaptive response. According to this line of reasoning, due to the presumed genetic pressure to overeat, it is probable to observe emotional eating even in the absence of individuals’ previous association between affective states and eating behaviors.

Similarly, the functionalist perspectives of happiness are likely to account for the positive association of happiness with food craving (Bongers et al., 2013). The functionalist perspective is a theoretical framework of emotions developed based on the assumption that emotions facilitate the unconscious pursuit of certain desire (Angit et al., 2011). In particular, functionalists suggest that the emotions of happiness are productive to the pursuit of self-improvement, which, in turn, encourages impulsive, indulgent, and hedonic consumption (Tanzer and Weyandt, 2019). For example, research has shown that the tendency toward emotional eating in response to feelings of happiness was prominent for female adults seeking weight loss (Bongers et al., 2013); the authors of this particular study suggested that happiness facilitates short-term and unconscious craving for hedonistic experiences, and this affective hunger often overrides individuals’ long-term goals (such as weight control, body fit, and disease prevention). The universal influences of happiness are then accounted by cultural, social, and contextual elements as eating is often associated with the celebration of events in spectator sports (i.e., the victorious and successful performances of supporting teams; Decrop and Derbaix, 2010).

The results of Experiment 2 are paralleled with the results of Experiment 1 given the non-significant associations between hope and food craving for both types of spectators (those who have had and those who have not had an association between emotions and eating behavior in the past). As aforementioned, the results support the functionalist perspective of emotions on regulatory behaviors (Godfrey et al., 2015); hope helps preserve individuals’ cognitive control capacity, and, thus, even in the face of a challenging situation, hopeful individuals are more likely to effectively manage situations-evoking emotions (i.e., high self-control; Stautz et al., 2018).

### Practical Implications

The results provide several important implications for marketers and media managers. First, from the perspective of marketing managers, the findings offer empirical and theoretical justification for managers’ propensity to utilize emotions-eliciting cues in promoting their products. For example, the Coca-Cola brand’s signature slogans, such as “Open Happiness” and “Taste the Feeling” as well as its commercials highlighting customers’ faces as they smile, and laugh are representative of strong, successful emotions-laden promotional strategies. Another example of a promotion built on similar high-quality strategies is the Google TV commercial that aired during the halftime segment of the Super Bowl game broadcast on February 2, 2020. Its broadcast during the middle of the show was meant to evoke feelings of sadness. In the commercial, the phrase “How to not forget” is being typed into a Google search bar; the rest of the commercial centers on an elderly man’s nostalgic voice as he asks Google Assistant for help to remember details about his late wife. Whether it is the laughter and smiles of the Coca-Cola promotional material or the nostalgia and melancholy evoked by the Google commercial, these strategies effectively activate viewers’ emotions to forge an emotional association with their product.

Given the results of the current study, marketing managers should proactively utilize their brand’s packaging and aesthetic design, slogans, and commentaries in advertisements to strategically align the marketing with particular emotions corresponding to game situations (i.e., the feelings of happiness in decisive victories and feelings of anger in close losses). As such, the current study helps managers develop context-dependent emotional positioning strategies. For example, at the Super Bowl LIV game, the Kansas City Chiefs (Kansas City, Missouri) competed against San Francisco 49ers (Santa Clara, California), and the Kansas City Chiefs emerged victorious. In this scenario, brand managers should then promote differently designed products across the two cities to trigger and boost their target markets’ emotional eating tendencies. Research in the field of design literature (e.g., Clark et al., 2021) suggests that yellowish color, decorative fonts, and curved shapes are more associated with feelings of happiness, while reddish color, hand script fonts, and angular shapes are more associated with feelings of anger.

Second, research suggests that individuals who have previous associations between emotions and eating are likely to show chronic tendencies of emotional eating (Altheimer and Urry, 2019), resulting in health risks such as severe obesity, diabetes, and heart disease (Nath et al., 2020). Media managers and legislators should then pay a closer attention to emotionally positioned advertising campaigns given that among the four types of discrete emotions examined, anger (experienced through close and decisive losses) has the greatest potential to evoke emotional eating. As an example of what this kind of campaign may look like in the real world, the “Like a Girl”’ campaign initiated by the company Always was designed to evoke feelings of anger as a means to attract attention and proactively encourage women to share their stories; in fact, the expression of “Like a Girl” is often used in teasing, mocking, or insult. However, even in cases where the idea behind a marketing campaign is informative and well intentioned, if the message is accompanied by the two game situations, anger-evoking campaign messages may translate into consumers’ emotional eating. Consequently, rather than becoming associated with the “Always” commercial, it would be more appropriate to be associated with Shopify’s “Autonomy” campaign highlighting the emotion of hope (the ad features motivational phrases such as “Start your journey” and “Get more out of life”). This recommendation is, therefore, for broadcast and media managers to strategically program their advertising and campaign schedules so that they align with the values of public health.

### Limitations and Future Suggestions

Caveats and recommendations should be addressed for future scholarship. First, and perhaps most importantly, the results of the current studies are limited given its exclusive reliance on self-reported measures of emotions, food consumption, and retrospective previous associations. For example, although the measure of previous association between emotions and eating might inherently rely on the retrospective nature of the behavior at hand, participants’ responses assessed *via* self-reported measures might have contained memory and recall bias, potentially compounding the results. Future studies then should extend the current experiments by employing alternative measures, such as the implicit association test (Greenwald et al., 2009; Chang et al., 2018), to explore individuals’ unconscious association between affect and food consumption in addition to utilizing other types of discrete emotions than those centered in this study.

Second, the strength of both sadness and fear was relatively and reasonably weak in Experiment 1 (compared to other types of emotions), conceivably rendering the researcher unable to produce fans’ food craving tendencies. Furthermore, for the sake of simplicity, Experiment 2 highlighted the three types of emotions (including happiness, anger, and hope) given the two game situations of decisive victories and close losses. The emotions of happiness and anger were designed in the current studies considering their significant explanatory power in accounting for food consumption evidenced in existing research (e.g., Macht, 1999); meanwhile, hope was selected because this emotion category is likely the most representative of regulatory and self-managed behaviors (Stautz et al., 2018). Nevertheless, there could be other types of emotions that would better explain individuals’ food craving behavior in the given context. In fact, recent studies have started to suggest that pride (vs. shame) is highly associated with feelings of hope in the context of spectator sports (Decrop and Derbaix, 2010; Chang, 2019), potentially causing aversive effects on food craving and obesity. Last, given the confined scope of this research (i.e., sports consumers, spectators, and fans), it would be limited to generalize the results to other contexts. Taken together, future studies may replicate the current studies using different emotion types along with their dynamic degrees of emotion intensity in a variety of emotions-laden consumption contexts (e.g., video gaming, movie and television viewership, and tourism) to enhance the generalizability of the findings.

## Data Availability Statement

The original contributions presented in the study are included in the article/supplementary material; further inquiries can be directed to the corresponding author.

## Ethics Statement

The studies involving human participants were reviewed and approved by University of Minnesota. The patients/participants provided their written informed consent to participate in this study.

## Author Contributions

The author confirms being the sole contributor of this work and has approved it for publication.

## Conflict of Interest

The author declares that the research was conducted in the absence of any commercial or financial relationships that could be construed as a potential conflict of interest.

## Publisher’s Note

All claims expressed in this article are solely those of the authors and do not necessarily represent those of their affiliated organizations, or those of the publisher, the editors and the reviewers. Any product that may be evaluated in this article, or claim that may be made by its manufacturer, is not guaranteed or endorsed by the publisher.

## Footnotes

^1^Given that food intake functions to narrow down human’s cognitive attention to immediate and pleasurable objectives (i.e., food), food craving under stressful circumstances is considered to be part of a fundamental aspect of human nature that seeks to reduce the consumer’s psychophysiological burden (Okumus and Ozturk, 2021).

^2^Food craving is defined as “an intense desire to consume a specific food” (Rodriguez-Martin and Meule, 2015, p. 1).

^3^ https://www.oculus.com
